# Measuring eating disorder attitudes and behaviors: a reliability generalization study

**DOI:** 10.1186/2050-2974-2-6

**Published:** 2014-03-10

**Authors:** David H Gleaves, Crystal A Pearson, Suman Ambwani, Leslie C Morey

**Affiliations:** 1 School of Psychology, Social Work and Social Policy, University of South Australia, Magill Campus, GPO Box 2471, Adelaide, SA 5001, Australia; 2 Texas A&M University, College Station, TX, USA; 3 Dickinson College, Carlisle, PA, USA

**Keywords:** Eating disorders, Assessment, Psychometric properties, Reliability generalization, Eating disorder inventory, Eating attitudes test

## Abstract

**Background:**

Although score reliability is a sample-dependent characteristic, researchers often only report reliability estimates from previous studies as justification for employing particular questionnaires in their research. The present study followed reliability generalization procedures to determine the mean score reliability of the Eating Disorder Inventory and its most commonly employed subscales (Drive for Thinness, Bulimia, and Body Dissatisfaction) and the Eating Attitudes Test as a way to better identify those characteristics that might impact score reliability.

**Methods:**

Published studies that used these measures were coded based on their reporting of reliability information and additional study characteristics that might influence score reliability.

**Results:**

Score reliability estimates were included in 26.15% of studies using the EDI and 36.28% of studies using the EAT. Mean Cronbach’s alphas for the EDI (total score = .91; subscales = .75 to .89), EAT-40 (total score = .81) and EAT-26 (total score = .86; subscales = .56 to .80) suggested variability in estimated internal consistency. Whereas some EDI subscales exhibited higher score reliability in clinical eating disorder samples than in nonclinical samples, other subscales did not exhibit these differences. Score reliability information for the EAT was primarily reported for nonclinical samples, making it difficult to characterize the effect of type of sample on these measures. However, there was a tendency for mean score reliability to be higher in the adult (vs. adolescent) samples and in female (vs. male) samples.

**Conclusions:**

Overall, this study highlights the importance of assessing and reporting internal consistency during every test administration because reliability is affected by characteristics of the participants being examined.

## Background

Although estimates for anorexia nervosa and bulimia nervosa approximate .3% and 1% respectively when using strict diagnostic criteria [1], disturbances in eating behavior and body image affect large numbers of individuals [2,3] and recent evidence suggests increases in the annual incidence of EDs in the U.K. [4] and a substantial rise in the point prevalence of ED behaviors in Australia [5]. Several measures are available for the assessment of ED symptomatology, but researchers or clinicians may falsely assume that these tools retain adequate psychometric properties such as internal consistency across all circumstances [6]. For instance, while reporting research results, authors frequently refer to the “reliability of a test”, a shorthand phrase that contributes to the misunderstanding by many researchers and students that tests, rather than scores, may be reliable [7]. This distinction between the reliability of test scores during a particular administration versus test reliability is significant; as emphasized by Wilkinson and the APA Task Force on Statistical Inference [8], “It is important to remember that a test is not reliable or unreliable. Reliability is a property of the scores on a test for a particular population of examinees” (p. 596) and thus reliability coefficients may vary depending on characteristics of the sample.

There are several reasons why it is important to examine and report reliability of test scores every time a measure is used. First, if score reliability is poor, the ability to measure the intended construct may be compromised, leading to a potential problem with validity of the data [9]; reliability of test scores is viewed theoretically as a necessary condition to establish validity, as “unreliable scores measure nothing” ( [9], p. 6). Second, poor score reliability may hinder the ability to find statistically, clinically, or practically significant effects [9]. When interpreting effect sizes, score reliability is an important factor to consider because measurement error impacts effect size [10-13], as a larger standard error contributes to a less precise effect size value [14]. Measurement errors cause observed effects to fluctuate across studies and may lead to underestimation of true effects [10]. This has led to recommendations for correcting effect size estimates for unreliable scores [15]. Third, total score variance affects reliability of the data set, and total score variance is impacted by characteristics of the participants [6,7]. Because score variability is a property of the data, reliability estimates will not remain constant across studies and should therefore be evaluated and reported as part of the process of describing the data.

Given the importance of test score reliability to scientific research, it is surprising that the editorial policies of journals often do not require this information to be reported and many authors do not report reliability estimates for their data [13,16]. Studies examining reporting rates for score reliability have estimates ranging from 7.5% for the Beck Depression Inventory [6] to 15.2% for the NEO Five-Factor Inventory [17], and Henson and Thompson [18] suggested that reporting rates are unlikely to exceed 40% for any test. Although reliability generalization studies have been conducted for self-report measures assessing various aspects of psychopathology, such as autism [19], substance abuse [20], depression [21], obsessive-compulsive symptoms [22] and general psychopathology [23], no published studies to date have evaluated the same for eating disorder symptoms.

Given the significance of accurately assessing test reliability, the present study employed reliability generalization (RG) procedures to report the mean score reliability for common measures of eating disorder symptoms and to examine variability in these estimates across sample characteristics. RG, a type of meta-analysis, characterizes the typical (i.e., mean) reliability of scores across studies, the amount of variability in reliability coefficients, and the sources of variability in reliability coefficients [16]. RG is consistent with previous work on validity generalization [24], in which researchers conduct analyses to determine if the validity of scores on a test was generalizable to different samples [12]. As with other types of meta-analysis, RG allows researchers to understand a large body of literature which may be producing inconsistent findings, in this case helping to understand differences in score reliability across multiple studies [6].

Two commonly used self-report measures of eating disordered attitudes and behaviors are the Eating Disorder Inventory (EDI; [25]) and the Eating Attitudes Test (EAT; [26]), both of which are available in revised forms (EDI-2; [27]; EAT-26; [28]). Research suggests that the EDI can distinguish individuals with AN [25] and BN [29] from nonclinical respondents. Conversely, the Eating Attitudes Test (EAT; [26]) assesses thoughts and behaviors related to anorexia nervosa and may be administered in the original 40-item version or a 26-item short form (EAT-26; [28]), both of which are typically highly correlated (*r* = .98; [30]). The EAT has also been shown to discriminate individuals with bulimia nervosa from control participants [29], eating disordered patients and controls, and binge eating patients from those with anorexia nervosa and bulimia nervosa [31].

An examination of the factors that influence reliability in eating disorder assessment can facilitate an understanding of how sample characteristics may contribute to variability in score quality. For example, other factors being equal, a heterogeneous set of participants will produce higher score reliability than a more homogenous group of participants [6]. Participant characteristics such as the type of sample, age and gender should be considered in evaluating score reliability. Other study factors potentially impacting test score reliability are sample size, type of reliability, culture/ethnicity, test format, test length, and test language. By identifying the conditions under which a test’s scores display higher or lower reliability, researchers will be able to tailor future studies about eating disordered attitudes and behaviors to conditions that will maximize score reliability and thereby yield additional control over one factor that influences effect sizes. Thus, in the present study we used RG procedures to study the mean score reliability for different versions of the EDI and the EAT to explore how score reliability of these eating disorder measures varies across studies, and explore the study characteristics that account for this variation.

## Methods

### Procedure

**Figure 1:**
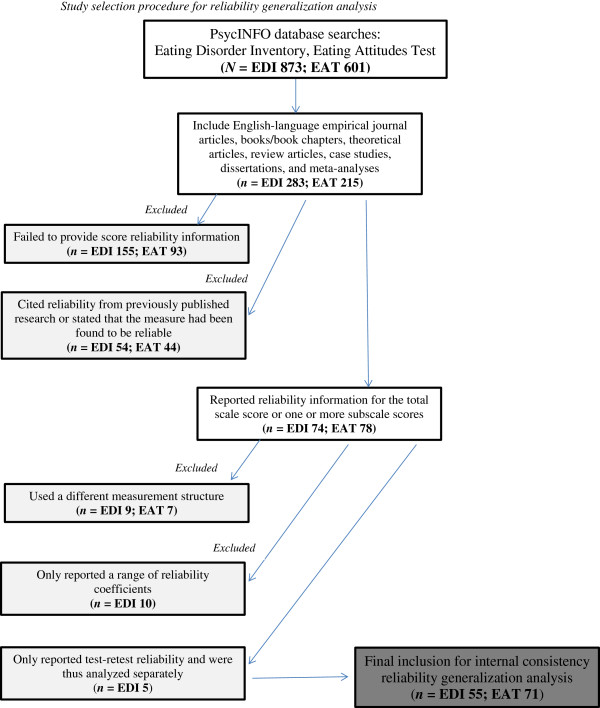
Study selection procedure for reliability generalization analysis.

The present study followed five steps for designing an RG study as recommended by Henson and Thompson [18]: selecting the measures to be analyzed, developing a coding sheet, collecting data, identifying potential dependent variables, and conducting analyses. For test selection, studies that utilized various forms of the EDI or the EAT were selected due to their common use as measures of eating disorder symptomatology in clinical and research settings. For developing a coding form and data collection, relevant reports were gathered through database searches of PsycINFO using the terms Eating Disorder Inventory and Eating Attitudes Test. The search period was from when the tests were first published (1979 for the EAT and 1983 for the EDI) through to the end of 2005, so approximately 27 years and 23 years of research publications for the EAT and EDI, respectively. The searches resulted in 873 references for the EDI and 601 for the EAT during that time period. Included references were published empirical journal articles; books/book chapters, theoretical articles, review articles, case studies, dissertations, and meta-analyses, articles not published in English were excluded from this study. Based on these criteria, 283 studies of the EDI and 215 studies of the EAT were reviewed and coded (see Figure 1 for a flow chart). The data coding sheet included codes for whether or not reliability information for the sample was reported and what type of reliability information was provided (i.e., internal consistency or stability). Additional study factors were also coded, including type of reliability coefficient reported, type of sample (clinical–eating disorder, clinical–general psychiatric, nonclinical, or mixed sample), type of study (treatment or other), age of participants, gender of participants, test language, test form, test length, and sample size. A single coder was used to code all studies because, unlike in more traditional meta-analyses, this study did not require any calculations to be made. Separate analyses were conducted using internal consistency and test-retest coefficients as dependent variables.

When using reliability estimates, some researchers combine Cronbach’s alpha [32] estimates with test-retest reliability estimates as a single dependent variable, but Dimitrov [33] cautioned against combining these estimates as they are not equivalent, and combining them together could lead to “mixing apples and oranges” ( [33], p. 792). Thus, after coding all the studies, we determined which study features were used in the analyses as independent variables based on whether enough data were available using that feature.

### Data analyses

SAS 8.2 software was used for all analyses, and published guidelines for conducting a reliability generalization study [18] and for conducting a meta-analysis [34] were followed. Overall mean reliability coefficients weighted by sample size were calculated for each measure, and sample-weighted mean reliability estimates broken down by predictor variables were also calculated. Sample size is one source of sampling error, with larger sample sizes providing more stable estimates of the population parameter because they are less susceptible to sampling error than smaller samples. Therefore, sample weighted means were used to reduce the effects of sampling error from smaller samples. If data were available, mean reliability coefficients are reported for the subscales of the measures. Additionally, the 95% confidence interval and percent of variance accounted for by sampling error were also calculated.

## Results

### Eating disorder inventory

In 155 (54.77%) out of 283 studies that used the EDI or EDI-2, the researchers did not provide any score reliability information (see Figure 1). In 54 (19.08%) studies, the researchers cited reliability estimates of scores from previously published studies or stated that the measure had been found to be reliable. The researchers reported reliability information for either the total scale score or one or more subscale scores for their sample in 74 (26.15%) studies; however, 10 of these studies were excluded from the analyses because the authors only reported a range of reliability coefficients for the subscale scores, and 9 studies were excluded for using a different measurement structure (see [25,27,29,31,35-87] for included studies). Five studies included only test-retest reliability and were analyzed separately. In some studies, researchers reported reliability information for more than one group of participants (e.g., control group and clinical group), resulting in more reliability coefficients for that scale than there were studies reporting score reliability information for that scale.

Table 1 presents the mean estimates of internal consistency for the EDI and its subscales and the means broken down by gender and age of participants, as well as type of sample and test language. All of the coded study factors could not be analyzed due to low variability or insufficient reporting of the characteristic. Only one of the articles reporting score reliability information was a treatment study, and the majority of authors did not provide sufficient information regarding participant ethnicity to allow for further analysis. Mean estimates of internal consistency for scores on the subscales ranged from .75 to .89 and for scores on the EDI, the mean estimate was .90. No studies reported estimates of internal consistency for the total score on the EDI-2. The Bulimia subscale had higher score reliability in clinical eating disorder samples than in nonclinical samples, whereas the Drive for Thinness and Body Dissatisfaction subscales did not display this difference. Mean estimates of score reliability also tended to be higher in the adult samples compared to the adolescent samples, as well as the female samples compared to the male samples.

**Table 1:** Mean internal consistency estimates for the eating disorders inventory (EDI)

		***Reliability***				
**Study characteristics**		**# of data points K**	**Total sample size N**	**Sample-weighted mean alpha (SD)**	**95% CI**	**PVA**_**SE**_
**EDI total**		**6**	**3269**	**.91 (.06)**	**.86 - .95**	**1.65%**
Gender:	Female	6	3269	.91 (.06)	.86 - .95	1.65%
Age:	Adult	4	995	.85 (.08)	.77 - .93	4.35%
	Adolescent	2	1137	.93 (<.01)	.93 - .93	100.00%
Type of sample:	Nonclinical	4	2989	.90 (.06)	.84 - .96	1.24%
	Clinical: Eating disorder	2	280	.95 (.01)	.93 - .96	99.32%
Test language:	English	4	995	.85 (.08)	.77 - .93	4.35%
	Non-English	2	2274	.93 (<.01)	.93 - .93	100.00%
**EDI body dissatisfaction subscale**		**55**	**22120**	**.89 (.04)**	**.88 - .90**	**6.51%**
Gender:	Female	44	17240	.90 (.03)	.89 - .91	9.82%
	Male	5	2443	.81 (.03)	.79 - .84	26.56%
	Mixed gender	6	2437	.90 (.03)	.88 - .92	11.02%
Age:	Adult	35	8311	.91 (.03)	.90 - .92	17.16%
	Adolescent	20	13809	.88 (.04)	.86 - .90	3.92%
Type of Sample:	Nonclinical	43	19844	.89 (.04)	.87 - .90	5.63%
	Clinical: Eating Disorder	11	2156	.90 (.03)	.89 - .92	19.05%
Test Language:	English	42	13557	.89 (.03)	.88 - .90	10.70%
	Non-English	13	8563	.88 (.05)	.85 - .91	3.17%
**EDI drive for thinness subscale**		**49**	**22335**	**.85 (.05)**	**.83 - .86**	**6.24%**
Gender:	Female	37	15831	.84 (.05)	.83 - .86	6.56%
	Male	4	2277	.79 (.03)	.77 - .82	37.17%
	Mixed gender	8	4227	.89 (.02)	.87 - .90	27.54%
Age:	Adult	32	7784	.86 (.04)	.85 - .88	15.07%
	Adolescent	17	14551	.84 (.06)	.81 - .86	3.29%
Type of sample:	Nonclinical	36	19788	.85 (.05)	.83 - .87	4.78%
	Clinical: Eating disorder	12	2427	.83 (.04)	.80 - .85	37.93%
Test language:	English	37	13943	.86 (.05)	.84 - .87	8.58%
	Non-English	12	8392	.83 (.06)	.80 - .86	4.17%
**EDI bulimia subscale**		**47**	**21875**	**.75 (.07)**	**.73 - .77**	**9.47%**
Gender:	Female	37	15905	.77 (.07)	.74 - .79	9.18%
	Male	3	2081	.67 (.02)	.65 - .69	100.00%
	Mixed gender	7	3889	.74 (.04)	.71 - .77	20.89%
Age:	Adult	31	7437	.81 (.07)	.79 - .83	11.55%
	Adolescent	16	14438	.72 (.04)	.70 - .74	14.17%
Type of sample:	Nonclinical	35	19861	.74 (.06)	.72 - .76	9.98%
	Clinical: Eating disorder	11	1894	.84 (.07)	.79 - .88	10.76%
Test language:	English	35	13483	.76 (.07)	.74 - .78	10.71%
	Non-English	12	8392	.74 (.06)	.70 - .77	7.57%

Note. CI = Confidence Interval. PVA_SE_ = Percent of variance accounted for by sampling error.

An examination of moderators of score reliability suggested that for the total EDI, there were some differences across adult vs. adolescent samples and clinical vs. nonclinical samples (see Table 1 for confidence intervals). For the most commonly employed EDI subscales (Body Dissatisfaction, Drive for Thinness, and Bulimia), mean estimates for internal consistency were .89, .85, and .75, respectively. For the Body Dissatisfaction subscale, the mean score reliability was higher in the female and mixed gender samples than in the male samples, and the adult samples had greater reliability than the adolescent samples. For the Drive for Thinness subscale, reliability was highest in the mixed gender samples followed by the female and male samples. For the other study characteristics, the adult and clinical samples displayed score reliability similar to their comparison samples, and the English language samples displayed greater reliability in their scores than the non-English test language samples. For the Bulimia subscale, the female and mixed gender samples displayed greater reliability than the male samples with the confidence interval for the male samples not including the means of the other two categories. The adult estimate was also greater than the score reliability estimate for the adolescent samples and the clinical eating disorder samples were greater than the nonclinical samples (see Table 1).

The percent of variance explained by sampling error varied widely for the EDI and its subscales, ranging from 1.65% to 100%. Generally, analyses conducted with a smaller number of data points frequently had a greater percentage of variance accounted for by sampling error. A smaller percentage of variance accounted for by sampling error suggests a greater percentage of variance is accounted for by true score variance across the observed studies.

### Eating attitudes test

Reliability information was not provided in 93 (43.26%) of 215 studies utilizing the EAT, and in 44 (20.47%) studies, the researchers made some reference to score reliability from previously published studies (see Figure 1 for a flow chart). Reliability information for either the total EAT score or one or more factor scores for their sample was reported in 78 (36.28%) studies. Seven of these studies were excluded from further analysis because the authors modified the measure or used different factors based on their own factor analysis of the EAT (see [26,28-30,69-135] for included studies). Results indicate that the sample-weighted mean estimates of internal consistency were .81 for the EAT-40 and .86 for the EAT-26. The mean estimates of internal consistency for scores on the EAT-26 factors were .80 for the Dieting factor, .67 for the Bulimia and Food Preoccupation factor, and .56 for the Oral Control factor. Table 2 presents the sample-weighted mean estimates of internal consistency for the EAT, as well as the means broken down by gender, age, type of sample, and test language.

**Table 2:** Mean internal consistency estimates for the eating attitudes test (EAT)

		***Reliability***				
**Study characteristics**		**# of data points K**	**Total sample size N**	**Sample-weighted mean alpha (SD)**	**95% CI**	**PVA**_**SE**_
**EAT-40**		**15**	**3925**	**.81 (.09)**	**.77 - .86**	**5.89%**
Gender:	Female	10	1950	.86 (.04)	.84 - .89	19.54%
	Male	2	492	.68 (.06)	.59 - .77	18.90%
	Mixed gender	3	1483	.79 (.08)	.70 - .88	4.55%
Age:	Adult	11	1855	.87 (.05)	.84 - .90	13.32%
	Adolescent	4	2070	.76 (.08)	.68 - .84	5.07%
Type of sample:	Nonclinical	12	3538	.80 (.08)	.75 - .85	6.45%
	Clinical: Eating disorder	2	193	.90 (.05)	.83 - .97	16.45%
Test language:	English	11	2833	.82 (.08)	.78 - .87	6.47%
	Non-English	4	1092	.78 (.10)	.69 - .88	5.72%
**EAT-26**		**54**	**11963**	**.86 (.05)**	**.84 - .87**	**11.50%**
Gender:	Female	42	9566	.85 (.05)	.83 - .87	11.06%
	Male	4	488	.85 (.03)	.82 - .88	72.61%
	Mixed gender	8	1909	.87 (.05)	.84 - .90	10.28%
Age:	Adult	42	9049	.86 (.05)	.85 - .88	12.89%
	Adolescent	11	2717	.85 (.05)	.82 - .88	11.94%
Type of sample:	Nonclinical	48	11321	.85 (.05)	.84 - .87	11.08%
	Clinical: Eating disorder	4	423	.90 (.02)	.89 - .92	100.00%
	Mixed clinical and nonclinical	2	219	.88 (.05)	.81 - .95	20.57%
Test language:	English	44	9077	.87 (.05)	.85 - .88	11.30%
	Non-English	10	2886	.81 (.04)	.79 - .84	25.69%
**EAT-26 dieting factor**		**24**	**10924**	**.80 (.07)**	**.77 - .83**	**5.50%**
Gender:	Female	22	10693	.80 (.07)	.77 - .83	5.06%
	Male	2	231	.81 (.03)	.77 - .85	100.00%
Age:	Adult	17	3870	.87 (.04)	.86 - .89	19.92%
	Adolescent	7	7054	.77 (.05)	.72 - .81	5.74%
Type of sample:	Nonclinical	22	10602	.80 (.07)	.77 - .83	5.27%
Test language:	English	21	10150	.80 (.07)	.77 - .83	5.35%
	Non-English	3	774	.86 (.04)	.81 - .90	17.23%
**EAT-26 bulimia and food preoccupation factor**		**23**	**10751**	**.67 (.11)**	**.63 - .71**	**5.44%**
Gender:	Female	21	10520	.67 (.11)	.62 - .72	5.12%
	Male	2	231	.71 (.11)	.56 - .87	17.26%
Age:	Adult	17	3870	.79 (.07)	.75 - .82	10.99%
	Adolescent	6	6881	.60 (.06)	.56 - .65	9.70%
Type of sample:	Nonclinical	21	10429	.67 (.11)	.62 - .71	5.29%
Test language:	English	20	9977	.67 (.11)	.62 - .72	4.92%
	Non-English	3	774	.71 (.04)	.67 - .76	60.81%
**EAT-26 oral control factor**		**18**	**4475**	**.56 (.10)**	**.51 - .60**	**21.02%**
Gender:	Female	16	4244	.56 (.10)	.51 - .61	19.03%
	Male	2	231	.60 (.02)	.57 - .63	100.00%
Age:	Adult	14	3065	.56 (.11)	.50 - .61	19.04%
	Adolescent	4	1410	.57 (.06)	.51 - .63	34.67%
Type of sample:	Nonclinical	16	4153	.55 (.08)	.51 - .59	27.63%
Test language:	English	15	3701	.54 (.10)	.50 - .59	21.52%
	Non-English	3	774	.63 (.04)	.59 - .67	100.00%

Note. CI = Confidence Interval. PVA_SE_ = Percent of variance accounted for by sampling error.

An examination of prospective moderators suggested that for the EAT-40, the female samples had higher reliability than the male and mixed gender samples, and the mixed gender group displayed higher reliability than the male group. However, with a small number of data points for analyses, it is important to interpret these comparisons with caution. The adult and clinical eating disorder samples displayed greater reliability than their respective comparison samples. For the EAT-26, the reliability was similar among the gender and age categories. The clinical eating disorder samples displayed greater reliability than the nonclinical samples, with the mixed clinical and nonclinical samples also displaying greater reliability than the nonclinical samples. The English-speaking samples also had a higher mean estimate of internal consistency than the non-English samples. Regarding subscale scores, female and male samples also displayed similar score reliability on the EAT-26 Dieting Factor. The adult samples had greater reliability than the adolescent samples, and the English test language samples had a higher mean estimate of reliability than the non-English samples. For the Bulimia and Food Preoccupation subscale, the mean estimate of reliability for the adult samples had higher reliability than the adolescent samples. For the Oral Control factor, the score reliability for the male samples was greater than the female samples, and the adolescent samples had a similar mean estimate of internal consistency as compared to the adult samples. The non-English language test samples had higher reliability than the English samples. Overall, for the EAT, the percentage of variance explained by sampling error ranged widely from approximately 5% to 100%. For the majority of the analyses, these values were less than 20%.

### Test-retest reliability analyses

Table 3 presents sample-weighted mean estimates of test-retest reliability for the EDI and EAT-26. For the EDI, the mean test-retest score reliability was .81, and for the EDI subscales, mean test-retest reliability estimates ranged from .42 to .77. The lowest mean test-retest reliability estimates were for the Drive for Thinness [60] and Bulimia [64] subscales. For the EAT-26, the mean test-retest reliability estimate was .87. However, due to the low number of data points available for each scale or subscale, these results should be interpreted with caution.

**Table 3:** Mean test-retest reliability estimates for the EDI and EAT

	***Reliability***				
**Study characteristics**	**# of data points K**	**Total sample size N**	**Sample-weighted mean reliability (SD)**	**95nnnnn CI**	**PVA**_**SE**_
EDI total	2	471	.81 (.06)	.72 - .90	12.68%
EDI body dissatisfaction subscale	3	256	.77 (.14)	.62 - .92	10.66%
EDI drive for thinness subscale	4	715	.60 (.15)	.45 - .75	10.07%
EDI bulimia subscale	4	715	.64 (.12)	.53 - .76	14.12%
EAT-26	4	920	.87 (.02)	.85 - .88	100.00%

Note. CI = Confidence Interval. PVA_SE_ = Percent of variance accounted for by sampling error.

## Discussion

This study used reliability generalization procedures to find the mean score reliability for different versions of the EDI and the EAT and to examine study characteristics (i.e., moderators) that might explain the variation in score reliability across studies. The reporting rate of score reliability information for the measures was higher (26.15% - 41.46%) than the reporting rate for other RG studies, such as the BDI (7.5%) [6] and the NEO (15.2%) [17]. However, given that score reliability information should be reported every time a measure is used, it is disappointing that such a large proportion of the studies using the EDI and the EAT failed to provide such information.

Overall, mean reliability estimates for the measures were acceptable, with only the Oral Control factor on the EAT-26 exhibiting questionable mean internal consistency [56]. For the EDI, the Bulimia subscale, which was designed to measure specific eating disorder attitudes and behaviors, displayed higher score reliability in clinical eating disorder samples than in nonclinical samples. Conversely, the Drive for Thinness and Body Dissatisfaction subscales exhibited similar score reliability in clinical and nonclinical groups. One potential explanation for this discrepancy is that the attitudes measured by the Body Dissatisfaction and Drive for Thinness subscales are common in both nonclinical and eating disorder samples, contributing to more reliable measurement of these attitudes across sample type. It is more difficult to characterize the effect of sample type on the two versions of the EAT because scores on these measures were primarily reported for nonclinical samples.

Regarding participant age and gender, mean score reliability tended to be slightly higher in the adult samples than in adolescent samples for all measures; however, reliability was generally acceptable in both groups. However, the EAT-26 Bulimia and Food Preoccupation subscale scores displayed mean reliability above .70 in the adult group but below .70 in the adolescent group. The higher reliability in the adult group may be expected as the measures were developed in adult samples. For all measures, there was a tendency for score reliability to be slightly higher in female samples than in the male samples, perhaps because eating disorder attitudes and behaviors are more prevalent among women resulting in greater score variability for this subpopulation.

Test-retest reliability analyses indicate that this type of reliability was generally acceptable for both measures, with the lowest estimate found for the EDI Drive for Thinness subscale, followed by the EDI Bulimia subscale. Although the EDI Bulimia scale did exhibit lower internal consistency estimates among certain samples, the EDI Drive for Thinness scale appeared to have similar score reliability across diverse samples; thus, it is somewhat surprising that this scale should exhibit the lowest overall test-retest reliability. One possibility for this might be that the construct assessed by this scale may be more subject to temporal fluctuations than some of the other constructs assessed by the EDI. Alternatively, the EDI Drive for Thinness scale is frequently employed in experimental studies involving brief interventions designed to change participants’ attitudes toward thinness (e.g., [136]), suggesting that this tool may be sensitive to fluctuations in drive for thinness. Finally, given the small amount of data available regarding test-retest reliability, these findings may be less statistically meaningful than the findings for internal consistency reliability estimates.

Although mean reliability estimates for scores on the EDI, EAT, and their subscales were generally acceptable, the data indicate that some of the subscales display greater score reliability in female, adult, and clinical (eating disorder) subpopulations, but with some variability across the different subscales. It is important that researchers measure internal consistency for their sample every time a measure is used as characteristics of the sample affect test score reliability. The present study demonstrates that reliability estimates do not remain constant across studies; therefore, researchers should ensure that the scores for their sample are found to be reliable as an initial step in any study. Examining and reporting test score reliability should be included as descriptive information about the data. Additionally, researchers can tailor future studies to maximize score reliability, which is one factor that influences effect sizes.

One limitation of this study is the small number of data points available for some analyses. Although information was reported for analyses where only 2–4 internal consistency estimates were available, these findings are less stable than a mean estimate based on 30–50 data points and therefore should be cautiously interpreted and were presented here only for the sake of completeness. Another potential limitation is having only one coder for the study. This decision was made because, unlike in traditional meta-analysis, the coder did not have to calculate effect sizes or other statistics and was only recording information as reported in articles; however, there is always the possibility that two coders could have disagreed about some of this basic information. In addition to addressing some of these design limitations and conducting a more recent search of the literature, future research could also examine the predictors of reliability estimates for other frequently employed assessment tools for eating symptomatology, such as the Eating Disorder Examination-Questionnaire [137].

## Conclusions

Reliability generalization is a valuable method of educating other researchers about reliability issues and emphasizing that reliability is “not an immutable unchanging property of tests” ( [18], p. 124). This study indicates that test score reliability for the EDI and EAT is greater for adult and clinical samples than for adolescent and nonclinical samples. Although it is important that disordered eating be reliably measured in an adult, clinical population, these findings are potentially troubling as it is also important that these concepts be reliably measurable in nonclinical adolescents who are at high risk for developing disordered eating attitudes and behaviors. In some cases, the differences in score reliability between adult and adolescent samples were small, and mean score reliability for adolescent samples remained acceptable overall. However, the differences between clinical eating disorder and nonclinical samples were generally larger. Without reliable measurement of these concepts in an at-risk adolescent population, researchers will have difficulty determining the true effectiveness of prevention programs designed to avoid or reduce future symptoms of eating disorders. Therefore, it is important for researchers to assess and report test score reliability with the measures they are using to determine the effectiveness of their programs.

## Competing interests

The authors declare that they have no competing interests.

## Authors’ contributions

DG developed the idea for the study, assisted with the research conceptualization and design; statistical analyses, writing and revising of the manuscript. CP obtained and coded articles for reliability generalization analyses, conducted analyses and drafted the manuscript from her doctoral dissertation under the supervision of DG and LM. SA assisted with the research conceptualization, literature research, and revising the manuscript. LM assisted with the design of the study and supervised article coding and statistical analyses. All authors read and approved the final manuscript.
